# The Effect of Different Storage Conditions on the Physical Properties of Pigmented Medical Grade I Silicone Maxillofacial Material

**DOI:** 10.1155/2013/582051

**Published:** 2013-03-28

**Authors:** Ayman A. Al-Dharrab, Seham B. Tayel, Mona H. Abodaya

**Affiliations:** ^1^Department of Oral and Maxillofacial Rehabilitation, Faculty of Dentistry, King Abdulaziz University, P.O. Box 80209, Jeddah 21589, Saudi Arabia; ^2^Faculty of Dentistry, Alexandria University, Alexandria 21534, Egypt

## Abstract

*Objective*. This study aimed to evaluate the effect of different storage solutions that simulate acidic, alkaline, and sebum conditiions on the physical properties of pigmented (colorant elastomer) cosmesil M511 maxillofacial prosthetic material. *Materials and Methods*. Sixty specimens were prepared according to the manufacturer's instructions and were tested before and after immersion of different storage conditions for six months at 37 °C. The following tests were performed: color changes (group I), solution absorption (group II), surface roughness (group III), and scanning electron microscopy (group IV). *Results*. There were no significant changes observed in the color and solution absorption tests while surface roughness revealed significant difference between control group and other testing storage medium groups, and this result was supported by SEM analysis that revealed limited surface changes. *Conclusions*. Cosmaseil material is an acceptable cross-linked formulation that withstands storage in different solutions with variable pH. The addition of pigment cannot vary the physical properties of these materials. Surface roughness test as well as SEM microscopic study showed moderate changes indicating a limited effect on the surface of the material.

## 1. Introduction

In contemporary society where beauty is considered essential, patients with facial mutilations due to congenital malformations, oncologic surgery, or trauma are often marginalized [1, 2]. In view of this reality, the goal of facial prosthetic technology is to offer individuals' aesthetic and comfort while improving their self-esteem and quality of life [3, 4].

Maxillofacial prostheses are used to transform congenital, developmental, and acquired defects of the head and neck into natural appearing reproductions of the missing parts, thus, providing an acceptable appearance and improved function. One of the Modern materials for external prostheses includes vinyl plastisols, polymethylmethacrylates, polyurethanes, latex, and silicone elastomers [5].

The prosthodontists are limited by the materials used in fabrication for facial prosthesis, movable tissue beds, graft and flap applications, unsuitability of anatomic undercuts, and patient acceptance toward the use of prosthesis [6]. There is no ideal facial prosthetic material, although there have been improvements in the last few decades, and silicone rubbers have established the current state-of-the-art material. Despite the advances in reconstructive and plastic surgery, replacement of the intricate facial structures is still required, especially the use of man-made materials as external prostheses [7].

Two major problems are associated with maxillofacial prostheses used to rehabilitate patients with extraoral-facial deformities, namely, degradation and discoloration. Deterioration is mainly caused by environmental exposure to ultraviolet (UV) light, air pollution, and changes in humidity. Facial prosthetics may absorb perspiration and sebum while resting on living human skin for extended periods. The absorption may cause changes in materials' structure, resulting in the deterioration of prosthesis. The human skin pH is 5.5 (mildly acidic). 

Sweat is a salty, watery solution produced by sweat glands. As sebum and sweat mix up on the skin surface, they form a protective layer that protects skin from “the elements” (such as wind or pollutants), also inhibits the growth of harmful bacteria and fungi. A recent research has shown that sebum secretion levels change in response to seasonal and environmental changes. The skin secretions, mouth rinse, and other solutions are also responsible for any color changes of the elastomeric prosthetic material. Twenty three hydrogen ion concentrations have a widespread effect on the function of the body's enzyme systems. An increase in skin surface pH encourages bacterial growth [8–10].

Silicone elastomers is the most common material used to fabricate maxillofacial prostheses because of its texture, strength, durability's and ease in handling, coloring, and patient, comfort. Chemically they are termed as polydimethyl siloxane they are of two basic types: room temperature vulcanizing (RTV silicone) and heat vulcanizing (HTV silicone) [11]. There are many advantageous characteristics of silicone prosthetics that consecrate silicone as the most suitable material for facial prostheses such as good biocompatibility and biodurability, wide service temperature range, nonadhesive properties, low toxicity, possible optical transparency, low chemical reactivity, and excellent resistance to attack by oxygen, ozone, and sunlight [12, 13].

Silicone elastomers are more color stable than other materials used in maxillofacial prostheses [14]. The physical and mechanical properties of silicone elastomer are dependent on the degree of cross-linking, the type and concentration of fillers in the elastomer network. For the degree of cross-linking, it depends on the nature and concentration of the thermal initiator, the fillers, the additives, and cure temperature and polymerization time [15]. The ideal elastomer-colorant combination should not only allow satisfactory esthetics to be achieved clinically, but should also maintain the esthetics and physical properties indefinitely, or at least until the patient's tissues have changed to the point that fit the prosthesis [16]. Realistic coloration of external facial prosthesis is an important feature for patient's satisfaction and acceptability. From the standpoint of attaining ideality for any extraoral prosthesis, it ranks high and indeed is the final emotional arbiter in successful rehabilitation. The base shade selected for a patient should be slightly lighter than the highest skin tones of the patient because the prosthesis will darken as the color is added. Cosmetic realism involves exacting replication of intrinsic (subdermal) colorant and extrinsic coloration [17].

Several techniques of color characterization include surface application of tinted silicone layers (which tended to peel with time), spray coloring of pigmented silicone elastomer with an artist's airbrush, incorporation of standard artist's oil paints below the surface of the prosthesis with a tattooing machine, mixture of earth pigments with silicone medical adhesive thinned with xylene and painted on the surface of the prosthesis. Recently, silicone pigmentation involves adding opacifiers to the base material [18, 19].

Pigments play the important role of imparting color to prostheses. Intrinsic coloration is longer lasting and is preferred but is more difficult to achieve. Since this issue is really important for maxillofacial research, so our study aimed to evaluate the effect of skin secretion on the physical properties of pigmented (colorant elastomeric) cosmaseil M511 maxillofacial prosthetic material after six months of immersion in different storage conditions at 37°C. 

## 2. Materials and Methods

### 2.1. Preparation of Test Specimens

**Figure 1:**
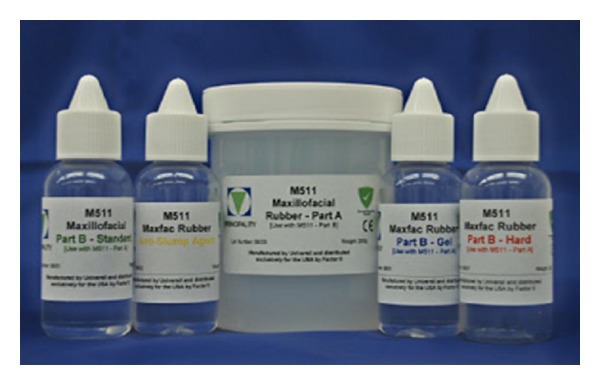
M511 silicon maxillofacial rubber material.

**Figure 2:**
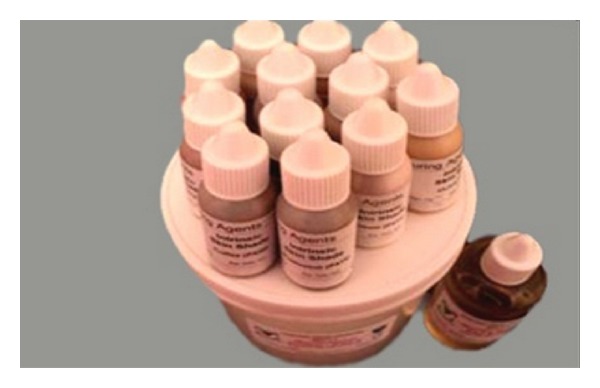
Cosmesil pigments coloring agents.

Sixty specimens were prepared according to the manufacturer's instructions as follows. The silicone materials (cosmesil series maxillofacial rubber M511, maxillofacial silicone system, HT platinum rubber, Medical grade Technovent Co, UK) (Figure 1). It is composed of dispersion fumed silica particles in platinum catalyzed (i.e., vinyl terminated, silicone fluid) were prepared by weighing the silicone elastomer (Base A) to a catalyst B using weight scale (Digital Electronic Weight Balance, OHAUS HP-320 OHAUS Corp., Florham Park, NJ, USA). To achieve a ratio of 10 : 1, that is, 10 g part A to 1 g part B = 11 g totally. They were mixed with the help of a white plastic spatula in a glass dish for two minutes until a homogenous mixture was obtained, then pigments (colorants intrinsic pigments-coloring agents, Product code: P409–P420,) (Figure 2) were added in amounts of 0.2% by weight [20, 21] and mixed until a homogenous color is obtained. The molds were coated with two applications of tinfoil substitute and allowed to dry. The mixture (elastomer-colorant combinations) then poured into the molds premade to the specific dimensions required by each International Standardization Specification. Molds then closed, clamped in the conventional way, and placed in a dry heat oven at 100°C for 1 hr. After polymerization, the specimens were carefully removed from the molds and flash was trimmed away with a sharp scalpel. All the specimens were left for 24 hours at room temperature after polymerization before grouping and testing.

The prepared specimens were divided into four groups with fifteen samples in each group according to the type of test specimens:

 group I: solutions' absorption test (15 specimens);
 group II: color stability test (15 specimens);
 group III: surface roughness (15);
 group VI: scanning electron microscope (15).

The specimens were serially numbered in each group, each specimen measured and considered as initial value, then the fifteen specimens were subdivided into 3 subgroups each of five immersed in storage conditions (the solutions (a), (b), and (c)) as in Table 1, in an incubator at 37°C for six months and measured again.

### 2.2. Laboratory Physical Tests

#### 2.2.1. Absorption Test of Group I

**Figure 3:**
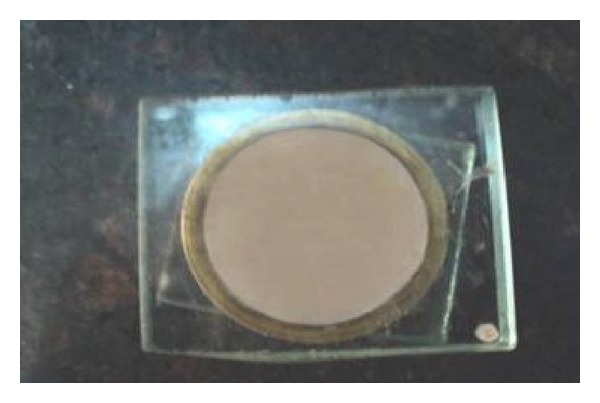
Ring mold.

**Figure 4:**
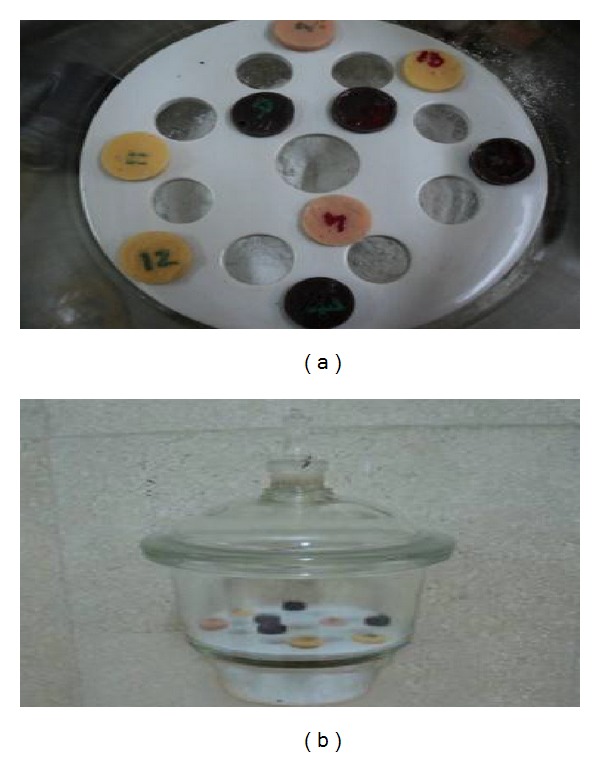
Absorption test specimens in the desiccators.

Fifteen disc shape specimens were prepared as previously described [22] using a copper ring mold with a circular hole of 25 mm diameter and 3 mm thick (Figure 3). The specimens were cured by the same method as described previously. The specimens were weighed initially (*w*_1_) by the help of digital electronic weight balance. The specimens of each subgroup were then placed in three separate glass screw topped jars, the first one containing the acidic solution (subgroup a), the second one in alkaline solution (subgroup b), and the third one in sebum solution (subgroup c). The glass jars were maintained in an incubator at 37°C for six months (24 hours per day), and then specimens were removed, blotted to remove excess solution, and reweighed again after the period of 6 months (*w*_2_). Specimens were then placed in desiccators containing phosphorus pentoxide and calcium chloride and re-weighed again (*w*_3_) (Figures 4(a) and 4(b)).

To calculate the amount of water absorption it was calculated by using the following equation:

(1) $$\begin{array}{c} \text{Absorption}\%=\frac{w_{2}-w_{3}}{w_{1}}, \end{array}$$

where *w*_1_ is the initial weight; *w*_2_ is the weight after absorption of water; and *w*_3_ is the weight after desiccation.

#### 2.2.2. Color Stability Test of Group II

**Figure 5:**
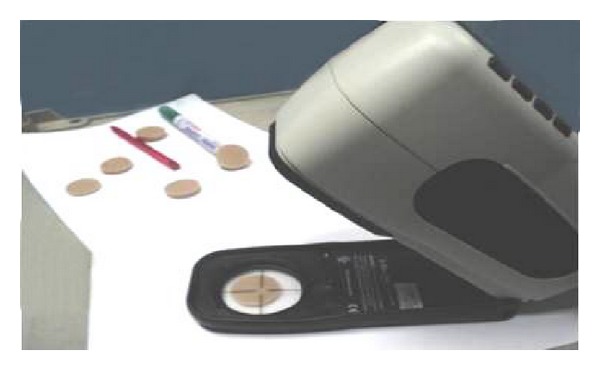
X-rite sphere spectrophotometer.

Fifteen disc shaped pigmented specimens (25 mm in diameter and 3 mm thick) were prepared as described previously. Color was measured with a portable sphere spectrophotometer (X-Rite, SP60 Series, USA) with a measuring head aperture of 4 mm in diameter. The specimens were placed on a white standard plate (calibration plate CR-A43). The Hunter Lab color scale was used to measure color. These values were carried out according to CIELAB systems (Figure 5) [23]. Using three dimensionless colorimetric parameters *L*, *a*, and *b*, whereby *L* indicates the brightness, *A* describes red-green content, and *B* describes yellow-green contents. Three readings were taken for each specimen, and mean values were calculated and recorded by the colorimetric. The color difference (Δ*E*) can be calculated by the following equation:

(2) $$\begin{array}{c} \Delta E={\left[{\left(\Delta L^{*}\right)}^{2}+{\left(\Delta a^{*}\right)}^{2}+{\left(\Delta b^{*}\right)}^{2}\right]}^{1/2}. \end{array}$$

The specimens was evaluated before immersion in the storage conditions and then after six months of storage.

#### 2.2.3. Surface Roughness Test of Group III

Roughness is the measure of the finer irregularities of surface texture that are inherent in the materials. Surface roughness average (Ra) is rated as the arithmetic average deviation of the surface valleys and peaks expressed in microinches or micrometers. If these deviations are large, the surface is rough; if they are small, the surface is smooth. For the surface roughness test, fifteen square shaped (25 mm × 25 mm and 3 mm thick) specimens prepared according to the American Society of Testing Materials (ASTM) [26] were measured before and after six months of immersion in storage solutions. A portable digital roughness tester was used (model Mahr Gmbh-Göttingen, Germany) with 0.01 *µ*m accuracy and 6 mm measurement course. For each specimen, 3 readings were done which, later, were transformed in mean values. The metallic matrix roughness was 0.6 Ra [27].

### 2.3. Scanning Electron Microscopy

SEM was performed for better investigation of the surface of the specimens before and after six months of immersion in the storage conditions. Cosmesil maxillofacial silicone elastomers M511 was monitored using a SEM (SEM: JSM-6360LA, JEOL, Tokyo, Japan). The sample preparation was achieved by fracturing a thin cross-section of test specimen, and then mounting on sample holders [28–30]. The specimen was sputter-coated with gold and the cross-sectional area was then observed at 2,500 and 5,000x magnifications.

### 2.4. Statistical Analysis of the Data

Data were entered to the computer using SPSS software package version 19.0. Quantitative data were described using mean and standard deviation for normally distributed data. The distributions of quantitative variables were tested. If it reveals normal data distribution, parametric tests were applied. If the data were abnormally distributed, nonparametric tests were used. For normally distributed data, comparison between different periods using ANOVA with repeated measures and post-hoc test was assessed using Bonferroni adjustment. 

Kruskal Wallis test was used to compare between different groups and post-hoc test was assessed using Mann-Whitney test. To compare between the different periods Friedman test was applied and Wilcox on signed ranks test with Bonferroni correction.

Significant test results are quoted as two-tailed probabilities. Significance of the obtained results was judged at the 5% level.

## 3. Results

Table 2 showed the mean values of percentage of solution absorption of pigmented M511 silicone maxillofacial material of subgroups (a, b, and c) which was not significant. Comparisons between each subgroup were not statistically significant.

The mean value of Δ*E* for color change of pigmented M511 silicone maxillofacial material for group II was showed in Table 3. Comparison between the control group before immersion and subgroups (a, b, and c) after immersion in different storage conditions was not statistically significant.

The mean value of surface roughness of the control group compared with subgroup (a, b, and c) (in Table 4) was statistically significant at 5% level. (^KW^ *P* = 0.001). Comparison within subgroup (a, b, and c) showed no significant difference. 

### 3.1. Scanning Electron Microscope for Group IV (SEM)

**Figure 6:**
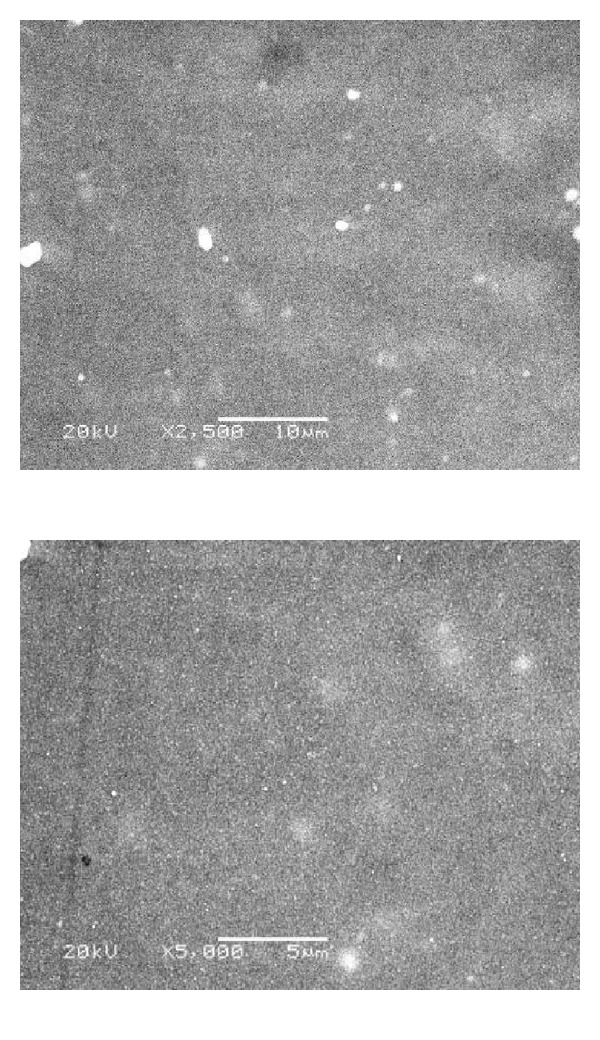
SEM micrograph showing smooth surface with few scattered fine silica of the control group (2500 and 5000x).

**Figure 7:**
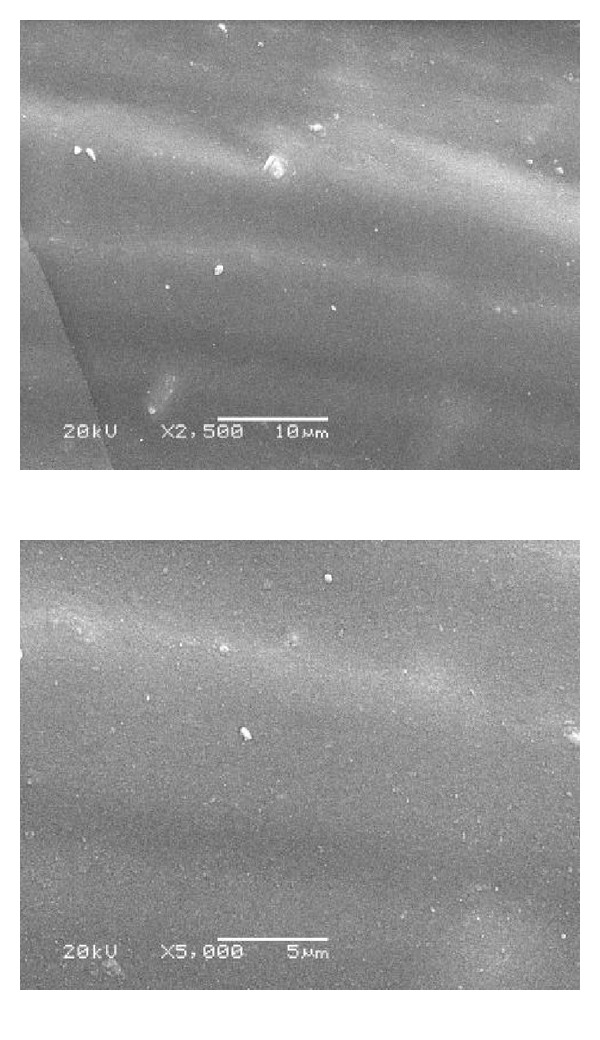
SEM micrograph showing rough surface with scattered fine silica particles of subgroup (a) after immersion in acidic solution (2500 and 5000x).

**Figure 8:**
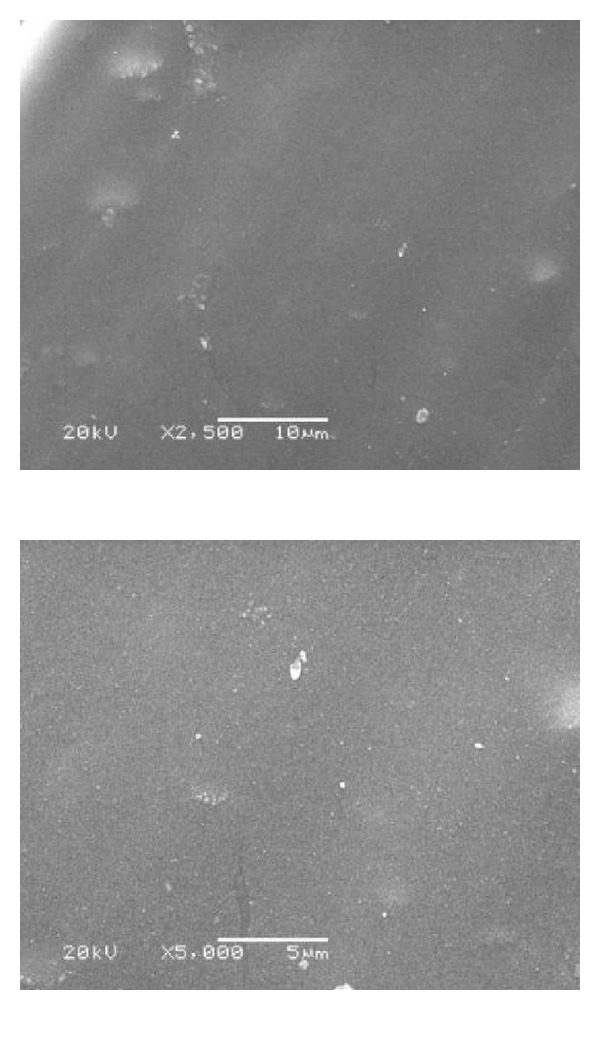
SEM micrograph showing smooth surface with some faint scattered vacuoles of subgroup (b) after immersion in alkaline solution (2500 and 5000x).

**Figure 9:**
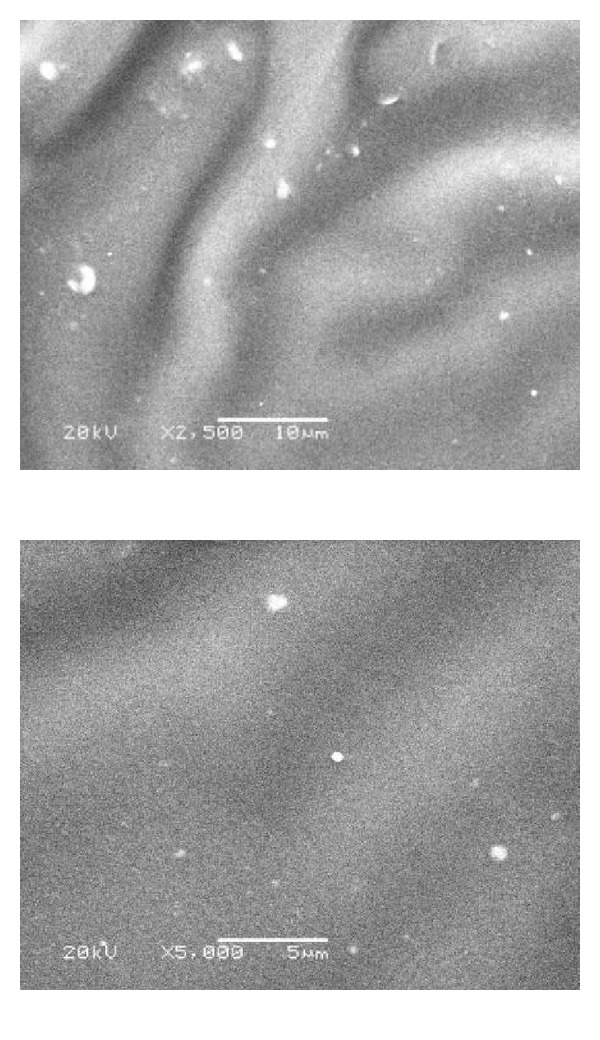
SEM micrograph showing wrinkled wavy surface with some scattered silica particles in subgroup (c) after immersion in sebum solution (2500 and 5000x).

Figures 6, 7, 8, and 9 revealed the scanning electron microscope micrograph for the cosmesil M511 material of the scattered groups and the different storage conditions (acidic, alkaline, and sebum) at resolution of 2500 and 5000x.

## 4. Discussion

Degradation of the color and the physical properties of maxillofacial prostheses in clinical use required refabrication approximately every 6 months. Ideally, the elastomer-colorant combination should not only allow satisfactory esthetics to be achieved clinically, but also to maintain the esthetics indefinitely, or at least until tissue changes in structure, color, or esthetics necessitate refabrication of the prosthesis. The color also should be stable over time and aging.

Cosmesil M511 maxillofacial silicone material was selected in our study because of its texture, strength, durability and ease in handling, coloring, and patient comfort [31, 32]. The M511 silicone was provided as a 2-part platinum (vinyl addition) cure system which is based primarily on a modified poly (dimethylsiloxane) structure, reinforcing silica, and fumed silica filler with a high surface area that maximizes the polymer/filler interactions. A platinum catalyst initiates the cross-link reaction. The cross-linking reactions in elastomers was catalyzed by a platinum complex that involves the addition of silyl hydride groups (–SH) in the silicone poly (dimethylsiloxane) to the vinyl groups (CH_2_=CH–) in the other silicones [28, 33].

Cosmesil skin shade kit (intrinsic pigments-coloring agents) of pigments suspended in silicone fluids was also used, to simulate the natural skin and ethnic skin tones and also leads to increased levels of color stability and pigment dispersion. The colors are suspended in a relatively thin fluid, and an agitator is incorporated into the dropper-type bottle to allow an easier redispersion of the powder throughout the fluid by simple shaking [34].

The success of any facial prosthesis depends on the physical and mechanical properties of the material used in its fabrication [35], therefore, solutions' absorption changes, color stability, surface roughness tests, and scanning microscope analysis were conducted to study the properties of M511 maxillofacial silicone material because of their clinical significance in fabricating facial prostheses

Solutions absorption and color stability were tested in this study as facial prosthetics may absorb perspiration (acidic and alkaline) and sebum while resting on living human skin for extended periods. The absorption may cause changes in materials' structure, resulting in the deterioration of prosthesis [36]. If we consider an average of 8 to 12 hours daily wearing of a facial prosthesis, then six months exposure period could be equivalent to 1 to 1.5 years of clinical service. The storage period in the solutions simulates 1.5 years of clinical service, which is enough for clinical application, since the mean lifetime of the prostheses is 14–24 months [20]. For this reasons a period of 6 months was selected that represents from 1 to 1.5 year of clinical use.

Percentage of solutions absorption after 6 months of storage in the simulated (acidic, alkaline, and sebum) was not significant in our results. This might be due to the presence of surface treated hydrophobic silica fillers present in the polymer matrix repelling water molecules and hence prevent solution absorption into the material. Moreover, the hydrophobic character of silicone matrix is enhanced, with the addition of this type of pigments because of the vinyl functional silanes groups present in their chemical structure, the vinyl additional cure system showed no bubbles, no shrinkage of final specimens, and the chemical double bond of vinyl group during cross-linking process had low polarity toward water, so there is no byproduct leading to absorption of any of these solutions [37]. This result was in agreement with Polyzois et al. [38] and Aziz et al. [22] but in contrast with Waters et al. [39] who found that facial prostheses may absorb saliva or sweat from surrounding facial tissue and also after washing the prosthesis in water which may affect the physical properties and also affect the perception of color matching to the surrounding facial tissue. 

The color change is one of the most important parameters when evaluating the performance of a facial prosthesis from a patient's perspective. For this study, the spectrophotometer was well suited for measuring the very small changes in the color of the elastomers, it has many advantages such as it being versatile: used for lab, or field operation; easy to read; quick color compare: permits quick measurement and comparison of two colors without need to create tolerances or store data; adds flexibility; with a rechargeable battery which allows for remote use [40, 41].

The results obtained in this study showed no significant color changes (ΔE*) in the tested specimens of control group and subgroups immersed in simulated perspiration, because silicone rubbers are resistant to salt solution and to dilute solutions of acids and bases and also resist absorbing organic materials. These results were in contrast with Yanagisawa [42] who investigated the effects of lipids on the color stability of MDX 4–4210 and silskin silicone elastomers using spectral transmittance and reflectance measurements. He reported that lipid absorption itself caused minimal changes in the samples, but the degeneration of the samples resulted from oxidation of the absorbed lipid. 

 Surface roughness was tested in this study because roughness is often a good predictor of the performance of a mechanical component, since irregularities in the surface may form nucleation sites for cracks or corrosion. The significant changes in the subgroups (a, b, and c) were not due to the polymer pigment physical interaction but due to the prolonged immersion in the different storage solutions (6 months) and rupture of chemical structure, formation of microcracks, and pits on the surface layer of material decrease the binding energy and surface energy; this gives rise to reduction of thermal withstand capability of surface layer which aggravates all other degradation effects [43]. The hydrophobicity of the material that has direct relationship to the surface appearance (smoothness), and the storage time factor had a significant influence on the material [28]. As supported by SEM analysis in Figures 7 to 9.

SEM examination was done to correlate the microstructure finding to the physical properties of the material. It gives us a magnified microimage of a surface of analyzed material. It resembles that viewing an object by electron microscope. The smooth surface observed in the control group may be attributed to the continuous polymerization process which promotes more complete polymeric chain making the silicone surface smoother with time, and the material had fumed silica particles that decrease the influence of absorbed solutions into cured materials and increase the hydrophobicity quality [28, 44]. After six months of immersion of the specimens in the different storage conditions (solution a, b, and c), the smooth silicone elastomer surface became rough with some irregularities in subgroups (a and c) due to small cracks surface with reduction in the hydrophobicity. This result might be due to acidic solution attacking the unsaturated macromolecules in the bulk of silicon and breaks them into small molecules or free terminal molecules (called radicals) which have tendency to react at free ends and may attach to water molecules in air; so they become hydrophilic as shown in Figure 7 [28, 45].

## 5. Conclusions

Cosmaseil maxillofacial silicone material is an acceptable cross-linked formulation that resists well storage in different solutions with variable pH. The addition of pigment cannot vary the physical properties of these materials. Continuous immersion of silicone specimens for 6 months revealed slight color changes as well as limited solution absorption.

Surface roughness test as well as SEM microscopic study showed moderate changes indicating that changes is limited to the surface of the samples.

Within the limitation of this study, patients wearing this type of silicone prosthesis are advised not to subject it to different solutions with variable pH especially during cleaning the prosthesis. 

## Figures and Tables

**Table 1:** The different storage solutions' composition.

Subgroup (a) Simulated acidic	Acidic perspiration (pH5.5) containing per liter of distilled water: 0.5 g L-histidine monohydrochloride monohydrate, 5 g sodium chloride, and 2.2 g sodium dihydrogen orthophosphate dehydrate.

Subgroup (b) Simulated alkaline	Alkaline perspiration (pH 8) containing the following per liter of distilled water: 0.5 g L-histidine monohydrochloride monohydrate, 5 g sodium chloride, and 5 g disodium hydrogen orthophosphate dodecahydrate.

Subgroup (c) Simulated sebum	Simulated sebum was prepared [24] using 10% palmitic acid and 2% tripalmitin dissolved in 88% linoleic acid (all wt %).

Both solutions (a) and (b) were prepared according to International Organization for Standardization (ISO) specification [25].

**Table 2:** The percentage of solutions' absorption of M511 silicone maxillofacial material of group I.

Parameter	Subgroups after immersion in storage condition		
	a	b	c
Mean ± SD	0.04 ± 0.02	0.03 ± 0.02	0.03 ± 0.02
^ Fr^ *P*		0.854	
*P*_ 1_		0.500	0.345
*P*_ 2_			0.465

Fr: Friedman test.

*P*_ 1_: *P* value for Wilcoxon signed ranks test between stage a and each other period.

*P*_ 2_: *P* value for Wilcoxon signed ranks test between stage b and c.

**Table 3:** The Δ*E* (SD) for color changes of group II of pigmented M511 maxillofacial silicone material.

	Control group before immersion	Subgroups after immersion in storage condition		
		a	b	c
Mean ± SD	14.86 ± 0.23	14.19 ± 0.33	14.13 ± 0.46	14.55 ± 0.81
*F*(*P*)		2.273 (0.132)		
Mean difference (*P*_1_)		↓0.674(0.293)	↓0.734 (0.072)	↓0.31 (1.000)
Mean difference (*P*_2_)			↓0.06 (1.000)	↑0.364 (1.000)
Mean difference (*P*_3_)				↑0.424 (1.000)

ANOVA with repeated measures test with the adjusted Bonferroni was assessed.

*P*_ 1: _stands for Bonferroni adjusted *P* value for comparison between before and each other period.

*P*_ 2:_ stands for Bonferroni adjusted *P* value for comparison between subgroup a and each other period.

*P*_ 3_: stands for Bonferroni adjusted *P* value for comparison stage b and c.

**Table 4:** Comparison of surface roughness mean value (Ra) for group III.

	Control group before immersion	Subgroups after immersion in storage condition		
		a	b	c
Mean ± SD	0.74 ± 0.11	2.90 ± 0.44	2.51 ± 0.95	1.65 ± 0.08
^ KW^ *P*		0.001*		
*P*_ 1_		0.009	0.009	0.009
*P*_ 2_			0.465	0.009
*P*_ 3_				0.028

^KW^ *P*: *P* value for Kruskal-Wallis test for comparing between the different studied groups.

*P*_ 1_: *P* value for Mann-Whitney test between stage and each other groups.

*P*_ 2_: *P* value for Mann-Whitney test between stage Basic and each other groups.

*P*_ 3_: *P* value for Mann-Whitney test between Sebum and Control.

#: Significant at *P* ≤ 0.008 using Bonferroni correction.
